# Chest radiography and computed tomography imaging in cystic fibrosis: current challenges and new perspectives

**DOI:** 10.1007/s00247-022-05522-4

**Published:** 2022-10-29

**Authors:** Pierluigi Ciet, Ronald Booij, Marcel Dijkshoorn, Marcel van Straten, Harm A. W. M. Tiddens

**Affiliations:** 1 Radiology & Nuclear Medicine Department, Pediatric Radiology Section, Erasmus MC-Sophia Children’s Hospital, Room Sb‑1650, Wytemaweg 80, 3015 CN Rotterdam, South‑Holland The Netherlands; 2 Department of Paediatric Pulmonology and Allergology, Erasmus MC-Sophia Children’s Hospital, Rotterdam, The Netherlands; 3 Department of Radiology & Nuclear Medicine, Erasmus MC, Dr. Molewaterplein 40, 3015 GD Rotterdam, South-Holland The Netherlands

**Keywords:** Chest, Children, Computed tomography, Cystic fibrosis, Lungs, Photon-counting computed tomography, Radiography

## Abstract

Imaging plays a pivotal role in the noninvasive assessment of cystic fibrosis (CF)-related lung damage, which remains the main cause of morbidity and mortality in children with CF. The development of new imaging techniques has significantly changed clinical practice, and advances in therapies have posed diagnostic and monitoring challenges. The authors summarise these challenges and offer new perspectives in the use of imaging for children with CF for both clinicians and radiologists. This article focuses on chest radiography and CT, which are the two main radiologic techniques used in most cystic fibrosis centres. Advantages and disadvantages of radiography and CT for imaging in CF are described, with attention to new developments in these techniques, such as the use of artificial intelligence (AI) image analysis strategies to improve the sensitivity of radiography and CT and the introduction of the photon-counting detector CT scanner to increase spatial resolution at no dose expense.

## Introduction

Cystic fibrosis (CF)-related lung disease remains the main cause of morbidity and mortality among people with CF [1]. Lung involvement in CF is characterised by chronic infection and inflammation with acute episodes of pulmonary exacerbation resulting in progressive diffuse lung damage and respiratory function decline [2]. In the last decade, new drug therapies and lung transplantation have led to a significant improvement in survival of people with CF [1]. Current median predicted survival for people with CF in the United States is 49 years (95% confidence interval [CI]=48.5–51.3 years) for those born in 2020 [3]. A longer life expectancy has been achieved thanks to the adoption of screening and yearly monitoring programmes, which aim to detect disease at a pre-symptomatic phase and allow timely treatment to prevent disease progression [1].

Current monitoring programmes rely on pulmonary function testing and imaging [4, 5]. The former is of limited use in preschool children, underestimates the early stages of structural CF lung disease and has limited predictive value in pulmonary exacerbation [4, 6]. For these reasons, imaging plays a pivotal role in the management of CF lung disease by providing structural regional information on the distribution and severity of the various abnormalities [7–9]. These lung abnormalities range from airwall thickening and bronchiectasis (early signs of irreversible lung damage) to fibrotic consolidation as seen in advanced stages of lung disease [10]. Mucus plugging and low-attenuation regions (either air-trapping or perfusion impairment) are linked to basic pathophysiology (hypoxic pulmonary vasoconstriction) and are potentially reversible with therapy [11]. Although most of these abnormalities are best depicted with chest CT, chest radiography is still being used by several CF centres to monitor disease progression [12]. Chest radiography has the advantages of being cheap and highly accessible and conveying the lowest amount of ionising radiation. Table 1 shows dose reference levels (DRLs) for chest radiography in children [13]. The latter is an important criterion in a large paediatric population such as that of people with CF. The introduction of low-dose and ultra-low-dose chest CT protocols has challenged the use of chest radiography, which has been replaced by CT at some specialised centres [14]. The use of CT instead of chest radiography has also been promoted by its higher sensitivity and specificity for early and subtle changes in CF lung disease [15]. Moreover, the development of automatic image analysis systems for chest CT in children with CF offers the clinician a convenient and objective tool to monitor disease progression [16]. Based on 25 years of experience at our centre, we give an overview about the current use of chest radiography and CT in the clinical routine of children with CF at our institution. We also present the latest technological developments, which are expected to significantly change imaging practice in children with CF.

**Table 1:** Age-dependent dose reference levels (DRL) for chest radiograph in children (derived from [13])

Examination	Age (year)					
	0	1	5	10	15	Adult
Chest radiograph projection (anteroposterior [AP] or posteroanterior [PA])	AP	PA	PA	PA	PA	PA
Tube voltage (kV)	70	73	75	80	80	86
Dose–area product (DAP) mGy∙cm^2^	3	5	10	20	40	90
E-60/DAP (mSv/Gy∙cm^2^)	2.2	0.58	0.41	0.28	0.16	0.15
Effective dose ICRP60 (mSv)	0.007	0.003	0.004	0.006	0.006	0.014

## Clinical scenario and imaging protocols

The use of each imaging technique in children with CF is strongly dependent on two factors: the age of the child and the indication for performing imaging [12]. A distinction is made between so-called uncooperative and cooperative children with CF, using as a cut-off the age of 6 years. An “uncooperative” child is defined as one who cannot follow breathing instructions, such as inspiratory and expiratory manoeuvres during the CT examination, either because of age (i.e. younger than 6 years) or level of consciousness (i.e. sedation).

Regarding the indications to perform CF imaging, three clinical scenarios are possible [12]. The first scenario is the initial imaging examination performed in children with CF for screening or first diagnosis. This group usually includes either young asymptomatic infants diagnosed by screening — sweat or genetic testing — or symptomatic patients, which may be a child or adult, the latter in the case of late diagnosis because of symptoms. The second clinical scenario includes people with CF undergoing routine (annual or biennial) clinical monitoring. In this group, a distinction should be made between patients with stable, declining or improving lung function [12]. The third scenario includes people with CF with acute respiratory symptoms caused by pulmonary exacerbation, which is often defined as rapid decline of lung function combined with increased respiratory symptoms [17].

**Fig. 1:**
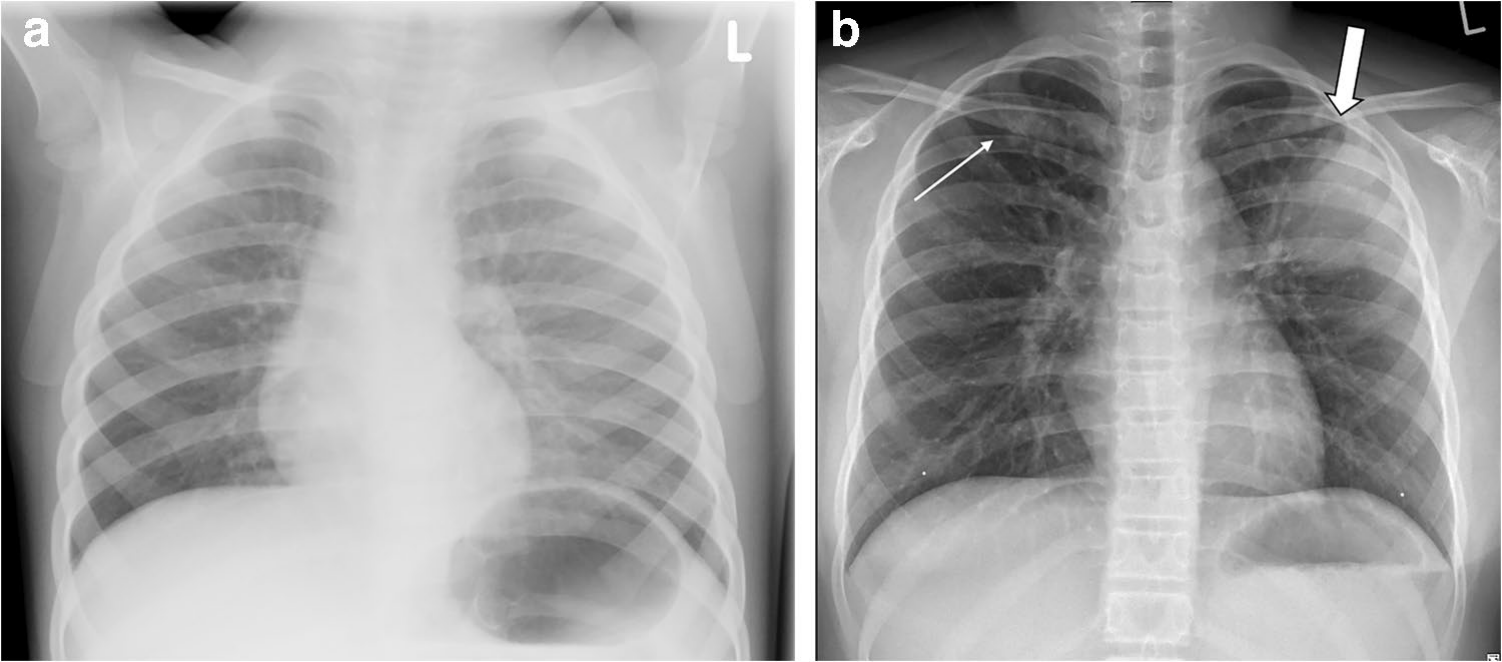
Chest radiography in children with cystic fibrosis and acute pulmonary exacerbation. **a, b** Anteroposterior (AP) chest radiograph in a 3-year-old boy (**a**) and posteroanterior (PA) chest radiograph in a 10-year-old boy (**b**). Note absence of clear abnormalities in (**a**). Conversely, (**b**) shows small peribronchial opacity in the right upper lung (*thin arrow*) and large consolidation in left upper lung (*thick arrow*)

For uncooperative children, chest radiography is usually performed as a single anteroposterior (AP) projection [5]. All cooperative CF subjects can receive a standard posteroanterior (PA) chest radiograph [5]. Figure 1 shows AP and PA chest radiographs in children with CF and pulmonary exacerbation. The lateral projection is usually not performed, both to reduce dose and due to the limited additional information yielded in the assessment of CF lung disease [5].

**Fig. 2:**
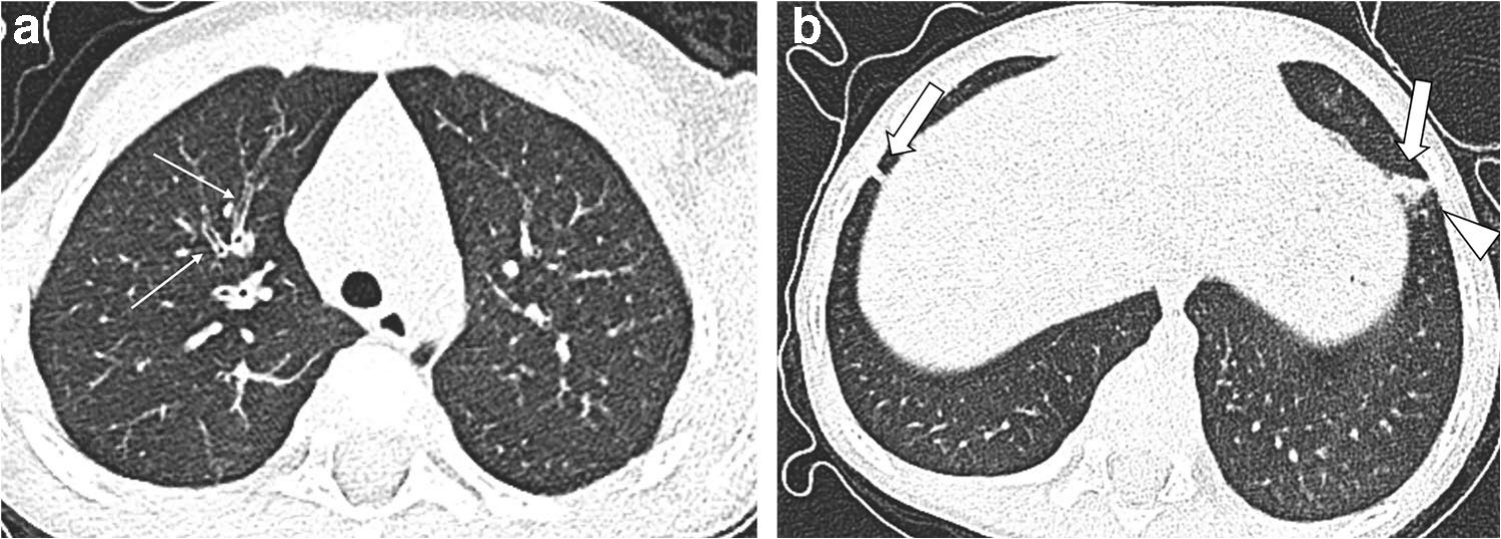
Acute pulmonary exacerbation in a 3-year-old boy. **a, b** Upper lobe (**a**) and lower lobe (**b**) sections of axial free-breathing CT without sedation show clear bronchial wall thickening (*thin arrows*) and focal parenchymal abnormalities in the lower lobes (*thick arrows*). The consolidation in the left lower lobe also shows a ground-glass halo (*arrowhead*), making it suspicious for acute respiratory infection

Similarly, in uncooperative children with CF, the recommended CT protocol consists of a free-breathing non-enhanced CT without sedation [18, 19]. Given the speed of the latest generation of CT scanners, the entire chest can be scanned in less than a second. For this reason, CT scanning can be performed without anaesthesia, thus avoiding cumbersome logistics and anaesthetic recovery times [20, 21]. When needed, young children can be immobilised in a vacuum mattress, providing consistent diagnostic image quality (Fig. 2).

**Fig. 3:**
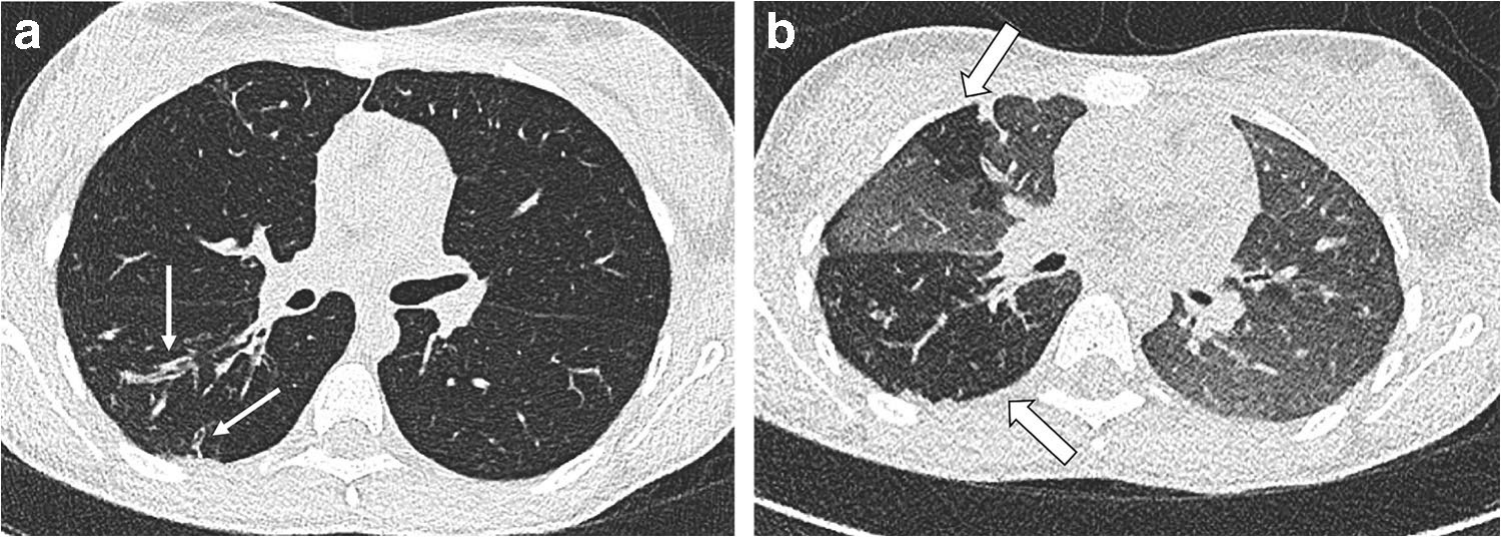
End-inspiratory and end-expiratory spirometry-guided non-enhanced CT in a 13-year-old girl with cystic fibrosis at her biennial follow-up. **a, b** End-inspiratory (**a**) and end-expiratory (**b**) axial CT images show clear bronchial wall thickening and bronchiectasis in the upper segment of the right lower lobe (*thin arrows*) and low-attenuation regions on the same area and also in the ventral part of the right upper lobe (*thick arrows*), indicating chronic small airways disease with trapped air and perfusion impairment

**Fig. 4:**
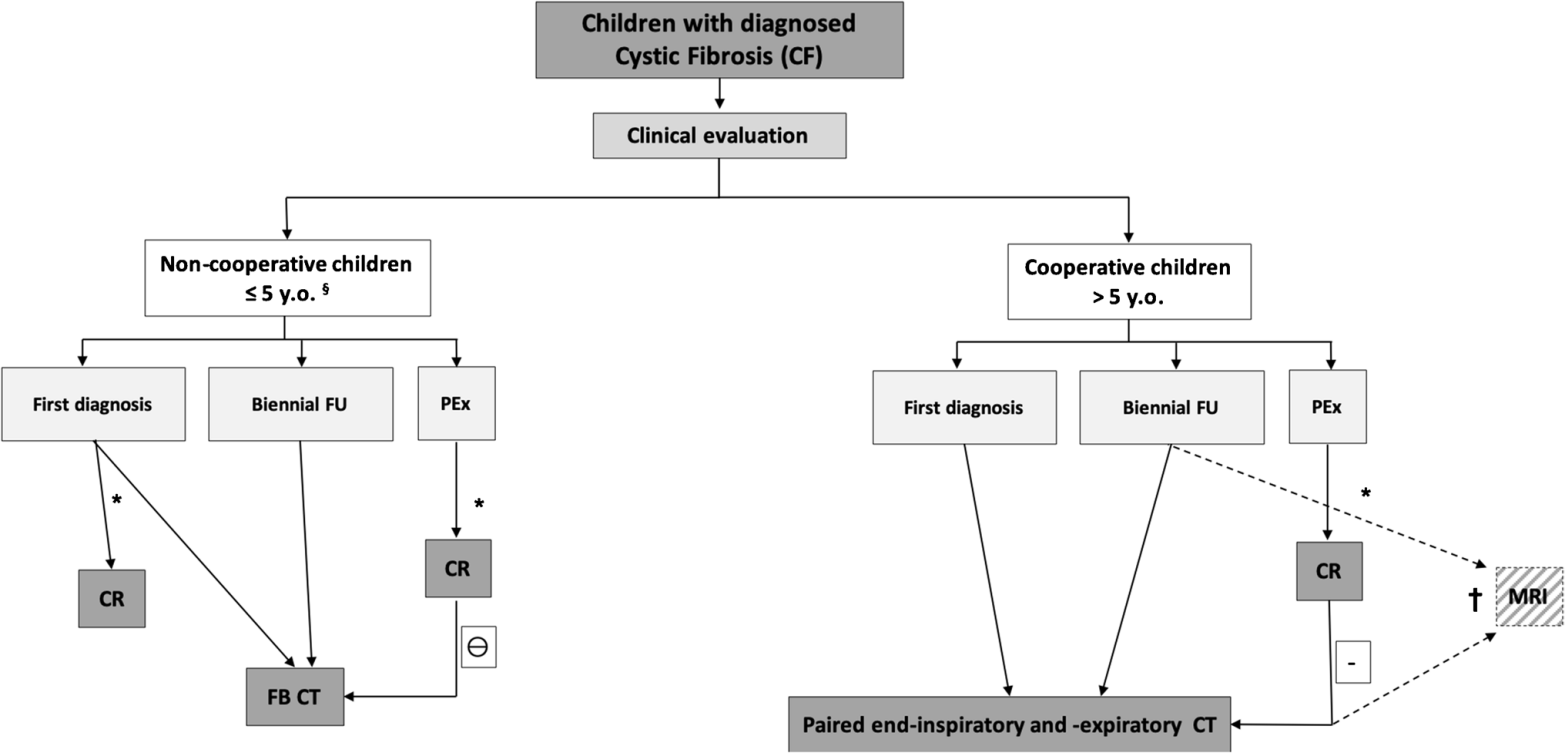
Current diagnostic algorithm for cystic fibrosis (CF) imaging. *CR* chest radiography, *CT* computed tomography, *FB* free breathing, *FU* follow-up, *MRI* magnetic resonance imaging, *PEx* pulmonary exacerbation, *y.o.* years old. The term “CT” in the flow chart is a general term, where dose should be set according to current dose reference level for paediatric chest CT imaging [24]. ^§^ Non-cooperative children are generally those who are younger than 5 years or mentally impaired, * depending on cystic fibrosis centre expertise, ⊖ CT can be performed in cases of negative chest radiography findings and persistent symptoms despite therapy, **†** at our centre we use MRI for short-term follow-up of pulmonary exacerbation and as follow-up technique in stable CF subjects in alternation with CT (1 year CT and the year after MRI)

In cooperative children with CF, we recommend performing an end-inspiratory and end-expiratory non-contrast-enhanced CT scan [22]. The expiratory scan at residual volume is important to assess trapped air, a major component of CF lung disease and an early sign of small airways pathology [23] (Fig. 3). At our institute, each paired inspiratory and expiratory CT examination is performed after training in supine position using a spirometer, which improves volume standardisation, image quality and contrast-to-noise ratio (CNR) for detecting trapped air in the end-expiratory scan [22]. The proposed diagnostic algorithm for chest radiography and CT, depending on the child’s age and clinical scenario, is presented in Fig. 4 [24]. This algorithm also includes cooperative children undergoing chest MRI. Although MRI is not a topic of this manuscript, it is an integral part of current CF imaging practice [12], including at our hospital.

## Using chest radiography for cystic fibrosis imaging: how we can do better

Several CF centres are still using chest radiography as the imaging technique of choice in infants and preschool children, although CT has shown higher sensitivity and specificity in detecting early abnormalities in both symptomatic and asymptomatic CF patients [15]. As mentioned in the introduction, this is mainly a result of radiography’s widespread availability, low cost and routine use by CF clinicians during patient follow-up.

**Fig. 5:**
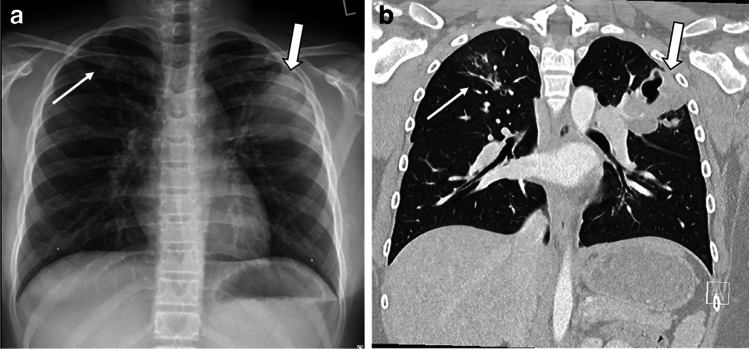
Acute pulmonary exacerbation in a 10-year-old boy. **a, b** Posteroanterior (PA) chest radiograph (**a**) and coronal end-inspiratory CT (**b**) show large cavitating consolidation in the left upper lobe (*thick arrow* in both). More subtle changes such as peribronchial opacities and mucus plugs in the right upper lobe are less visible on chest radiograph (*thin arrow* in **a**) compared to CT (*thin arrow* in **b**)

The sensitivity of chest radiography is poor, and the variability among radiologists in interpreting chest radiography is high, even when combined with adequate scoring systems [25, 26]. Furthermore, CT contributes significantly to clinical decision-making in contrast to chest radiography [15]. A recent study showed that CT is a simple stratification tool that can identify children at increased risk of poor outcomes in later life and is an early indicator of the effectiveness of interventions in very young children who have disease-modifying potential [23]. Therefore, using chest radiography in the early phase of CF lung disease seems to be of limited value, with CT being a more efficient way of detecting early disease and monitoring disease progression [12]. The use of chest radiography is also debated during pulmonary exacerbation, especially in children with advanced disease [27]. Children with CF and advanced lung disease are at higher risk of pulmonary exacerbation, and on chest radiography it is more difficult to detect any new radiographic change in a lung substrate with diffuse parenchymal abnormality [27] (Fig. 5).

Recent national guidelines have challenged the current use of chest radiography for follow-up of people with CF, especially in the early phase of the disease [12]. A step forward would be improving the sensitivity of chest radiography by applying automatic artificial intelligence algorithms. Preliminary studies showed that an AI-based algorithm could be used to perform an automated Brasfield scoring of chest radiographs and perform similarly to a paediatric radiologist, allowing for an objective and automatic assessment of radiographs for CF imaging [28]. These AI solutions could potentially reduce diagnostic uncertainty.

## From low-dose to ultra-low-dose computed tomography

The main limitation of CT for routine imaging of people with CF, especially children, is the exposure to ionising radiation. Dose has been progressively lowered thanks to technical advances and awareness created by the introduction of dedicated dose reference levels (DRL) [29] and by campaigns such as Image Gently [30]. However, a significant heterogeneity in protocols remains, with a lack of consensus relative to the optimal timing of the first CT examination and the dose level [12]. Current best clinical imaging practice in several CF centres within the European Cystic Fibrosis Clinical Trial Network is to perform a non-enhanced CT biennially (once every 2 years) with a radiation dose as low as reasonably achievable (the ALARA concept), while avoiding sedation [31]. The risk related to the cumulative dose conveyed with this follow-up CT scheme (total of nine CTs from ages 1 to 17 years) has been deemed reasonably low [32]. Moreover, some studies have shown that chest CT can be performed at largely reduced radiation exposure levels [33, 34] without significant loss in image quality. Tables 2 and 3 provide dose ranges for low- and ultra-low-dose CT for CF imaging [35, 36]. Further dose reductions are expected by the introduction of the new photon-counting detector CT (PCD-CT) scanner, which can provide a dose reduction of 30–60% at no expense to image resolution [37]. More details related to the advantages of PCD-CT for paediatric chest imaging are given in the following section.

**Table 2:** Age- and weight-dependent reference levels for low-dose chest CT imaging in children from our institution with cystic fibrosis

Patient age (years)	Patient weight (kg)	Mean CTDI_vol_, 32-cm inspiratory scan (mGy)	Mean CTDI_vol_, 32-cm expiratory scan (mGy)	Total mean CTDI_vol_, 32-cm (mGy)	Irradiated length (cm)^a^	DLP (mGy∙cm)	Conversion factor^a^	Effective dose for inspiratory scan (mSv)	Tube current (mA)	Tube voltage (kV)	Average pitch
0	4	0.64	0.32	0.96	7.56	4.84	0.07	0.4	CARE Dose4D (Siemens Healthineers)	CARE kV (Siemens Healthineers)	1.55
1	10	0.74	0.37	1.12	10.75	7.96	0.05	0.4			
5	18	1	0.50	1.50	14.17	14.17	0.03	0.5			
10	33	1.49	0.74	2.23	17.75	26.45	0.02	0.6			
15	54	2.08	1.04	3.12	n/a	n/a	n/a	n/a			
18	58	2.2	1.10	3.30	22.56	49.63	0.01	0.7			
20	65	2.36	1.18	3.54							
75	2.56	1.28	3.85								
110	3.46	1.73	5.19								

*CTDI*_*vol*_ volumetric computed tomography dose index for 32-cm phantom, *DLP* dose–length product, *n/a* not available

^a^Irradiated length and conversion factor as described by [35]

**Table 3:** Ultra-low-dose chest CT dose reference

Mean age (years)	Mean weight [SD] (kg)	Mean CTDI_vol_, 32-cm inspiratory scan (mGy)	Mean effective dose (mSv)	Tube current (mA)	Tube voltage (kV)	Average pitch
21.6	51.26 [19.7]	N/A	0.08	20	80	1.38

Table derived from [36]

*CTDI*_*vol*_ volumetric computed tomography dose index for 32-cm phantom, *N/A* not available, *SD* standard deviation

Further dose reductions could be achieved by introducing a patient-tailored CT imaging follow-up scheme that would stratify children with CF according to their risk factors for disease progression. Factors used for risk stratification include chronic bacterial infection, pulmonary exacerbation rate, pancreatic insufficiency, nutritional state, age at diagnosis, therapy adherence and use of new CF transmembrane receptor (CFTR) modulators [12]. Using this risk stratification method, imaging intervals could be modified according to disease status, with longer CT scan intervals in more stable CF patients and consequent reduction of cumulative dose [12]. This approach is particularly important in the new era of CFTR modulation therapy, which has shown remarkable efficiency in terms of improving lung function and reducing structural abnormalities [38].

## Photon-counting detector computed tomography: a potential game changer in paediatric chest imaging

**Fig. 6:**
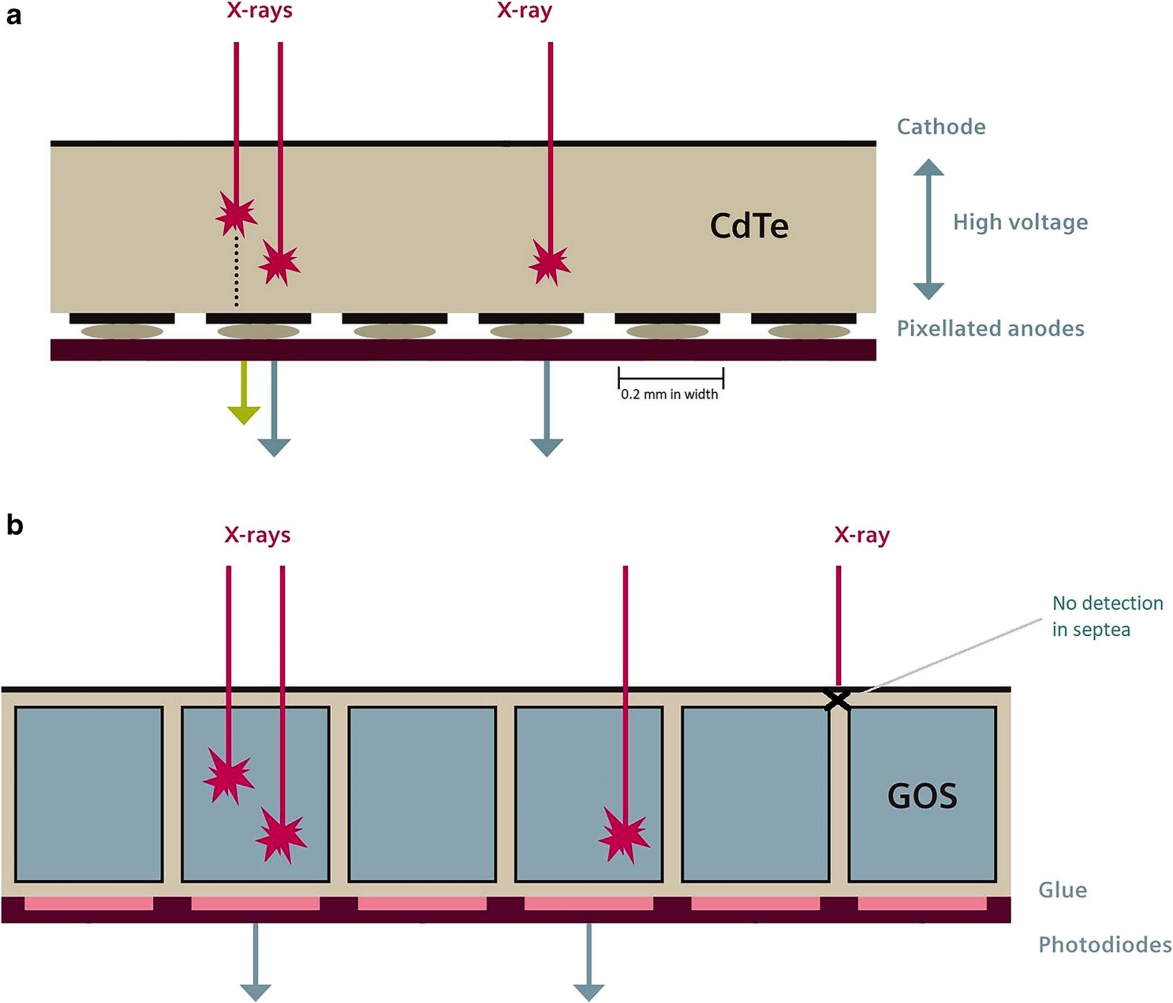
Illustration of photon-counting detector CT (PCD-CT) and conventional energy integrating detector CT (EID-CT) scanners. **a, b** The current EID (**b**) and PCD (**a**) CT scanners with improved detector efficiency and more detailed information thanks to direct conversion of the radiation signal, smaller detector elements and spectral information (intensity and energy) obtained by counting the number of incoming photons per energy bin. *CdTe* cadmium telluride, *GOS* gadolinium oxysulfide. Images adapted from [39]

Photon-counting detector CT (PCD-CT) scanners have been recently introduced in the market as the greatest new technological innovation in CT imaging [37]. PCD-CT enables higher resolution than standard detectors because PCD-CT detector pixels can be made smaller given that a separate layer to convert X-rays into light is not required. The PCD-CT detector consists of a single semiconductor layer. Therefore, X-ray conversion to electric signal is direct and more efficient than with conventional detectors (Fig. 6) [37].

**Fig. 7:**
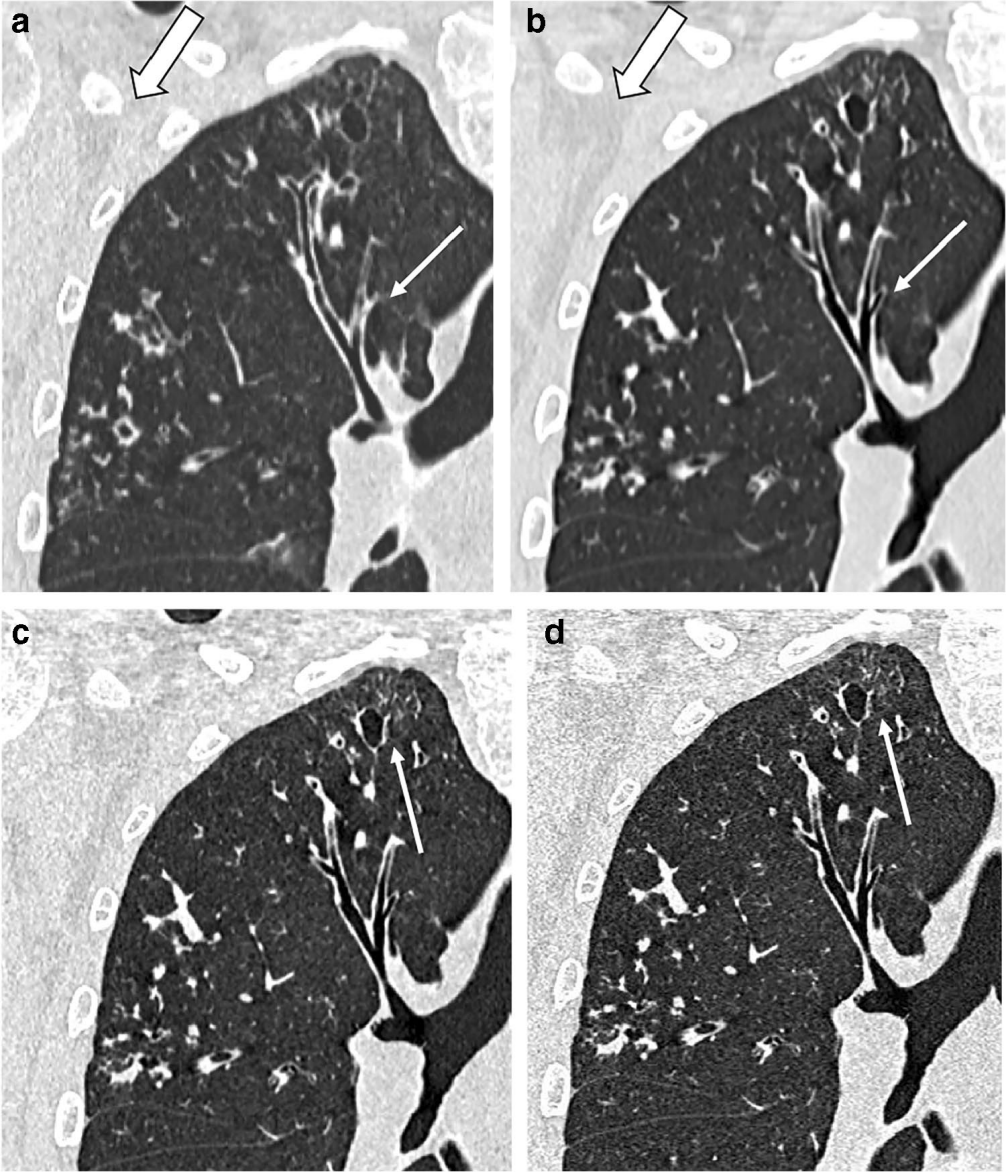
Photon-counting detector CT (PCD-CT) and conventional energy integrating detector CT (EID-CT) scanning in a 20-year-old man with cystic fibrosis. **a–d** Coronal detail of end-inspiratory CT using conventional EID-CT (**a**) and PCD-CT (**b–d**). Images (**a**) and (**b**) have slice thickness of 1 mm, and images (**c**) and (**d**), 0.6 mm and 0.2 mm, respectively. All four images have similar kernels including iterative reconstruction. Comparison of images (**a**) and (**b**) shows a clear reduction of image noise (reduced granularity in the axillary region, *thick arrow*) and sharper definition of bronchial wall and cystic bronchiectasis in the PCD-CT (*thin arrow*). In images (**c**) and (**d**), the increased resolution allows sharper details of the most peripheral structures (*thin arrow*)

Because energy integrating detectors measure the total absorbed X-ray energy, high-energy photons contribute relatively more to the total signal than low-energy protons. This results in a poorer contrast-to-noise ratio (CNR) because the tissue contrast is low at high energies [37, 39]. CNR is improved with PCD-CT, where higher weight can be assigned to low-energy photons, improving contrast between the tissues [37]. Moreover, PCD-CT scanners have a higher dose efficiency than energy integrating detector CT (EID-CT), mainly because of electronic noise suppression, especially for low-dose imaging. At low intensities, such as in low-dose imaging, electronic noise can substantially degrade image quality. Recent tests on lung phantoms using low-dose chest CT protocols showed up to 20% reduction in image noise [37] (Fig. 6). This would be particularly advantageous for paediatric imaging, where imaging protocols are usually at a lower dose than in adults [40]. Another important advantage of PCD-CT scanning in paediatric imaging is its much higher spatial resolution as compared to EID-CT [37]. The PCD-CT scanner at our institution enables acquisition of CT data with the smallest detector pixel setting of 0.2×0.2 mm^2^ compared to the EID-CT scanner of 0.6×0.6 mm^2^. This higher resolution is particularly important in younger children, where the sizes of airways and vessels are much smaller than in adults [40]. A recent review highlighted the great potential of PCD-CT to improve detection of lung abnormality thanks to its higher-resolution, lower-noise, low-dose imaging and spectral imaging capabilities (Fig. 7) [40]. Finally, PCD-CT can potentially further reduce the dose of chest CT imaging. The noise conveyed by PCD-CT is lower at the same level of radiation exposure than that of conventional CT scanners as a result of both minimised electronic noise and optimal X-ray photon-energy weighting [37]. Recent in vivo human experiments confirmed a dose reduction of up to 34% in photon-counting CT scans of the chest [37, 41]. Moreover, the improved spatial resolution of PCD-CT could be used to lower radiation dose at similar spatial resolutions and noise levels of the current protocol. Thus, it is possible that PCD-CT might reduce radiation levels by approximately 30–60%, depending on the imaging task [37]. This could bring CT imaging into a new era of ultra- or hyper-low-dose imaging, where the justification for chest CT is not hampered by the higher radiation exposure relative to chest radiography.

## Moving from qualitative to quantitative imaging

**Fig. 8:**
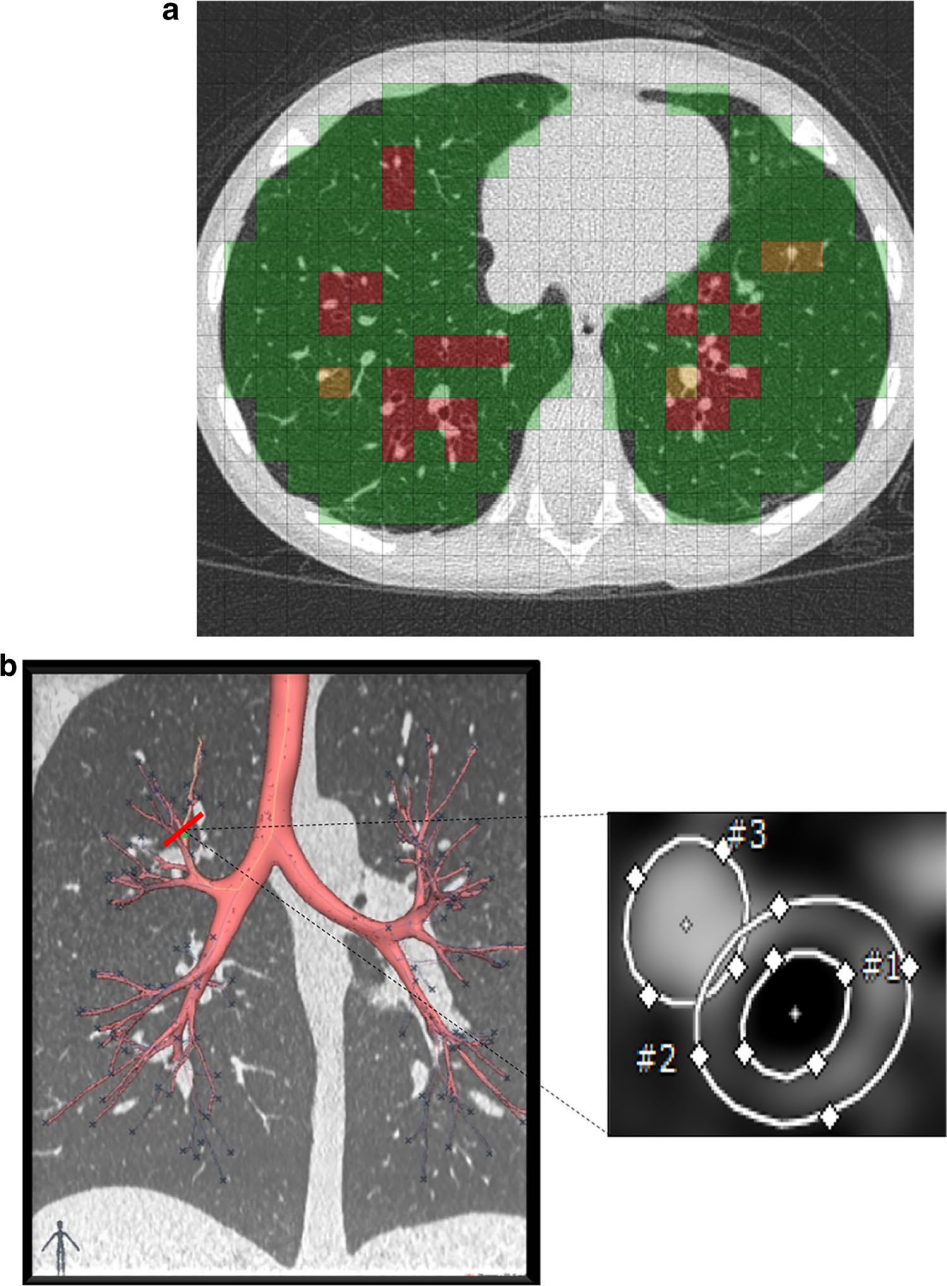
Advanced image analysis of CT in a 12-year-old boy with cystic fibrosis (CF). **a, b** PRAGMA (Perth-Rotterdam Annotated Grid Morphometric Analysis) CF score (**a**) and airway–artery (AA) ratio (**b**). The PRAGMA-CF score is a scoring system that uses a grid to quantify all typical CF lung disease components. In the example, green colour is used for normal lung tissue, red for bronchiectasis and yellow for bronchial wall thickening. The percentage of disease (%Dis) is the total score of these components expressed as a percentage (%) of the total lung volume. In the AA ratio, the entire bronchial tree is automatically segmented (**b**) along with the adjacent pulmonary arteries. Each AA pair is then segmented (perpendicular view of airway and artery measurements) to compute several parameters, such as percentage of bronchiectasis, bronchial wall thickening and lack of tapering. Both PRAGMA-CF and the AA method have been automated using artificial intelligence techniques and are validated

Artificial intelligence and machine learning solutions have been tested and validated for CT imaging in people with CF [8, 23]. AI-based image analysis software further increases sensitivity of CT for detecting early signs of CF lung disease and for monitoring disease progression in both symptomatic and asymptomatic patients [8, 38, 42]. AI-based CT outcome measures can be used in clinical trials for the evaluation of novel treatments [42]. These AI-based image analysis tools for chest CT provide reader-independent quantitative outcomes such as airway–artery diameter ratios, airways tapering indices and trapped air volume [8, 38, 42] (Fig. 8). Convincing results from multi-centre studies using a fully automated commercially available AI-based image analysis system [38] and the upcoming introduction of commercially available software for CF CT imaging [16] are expected to change clinical trial design and clinical practice. Paediatric radiologists need to have in-depth knowledge of these new tools to be able to interpret their results for providing an objective assessment of CF lung disease status to the clinician [14].

## Summary

In the last decade the use of chest radiography and CT for CF imaging has changed. The introduction of new disruptive technology, such as PCD-CT, is expected to improve image quality even further and drastically reduce the radiation exposure of CT, making chest radiography an obsolete radiologic tool for CF lung disease monitoring. Chest radiography may survive the challenge of CT if its sensitivity can be enhanced by dedicated automatic AI-based scoring systems.

The possibility of combining an ultra- or hyper-low-dose protocol to automatic image analysis systems for CT in CF imaging will have a major impact on clinical practice and expand the role of the radiologist to include being the gatekeeper of these technologies. The new challenge for CF imaging is likely to be tailoring the follow-up imaging monitoring scheme in children with CF using established CF-related risk factors.
